# Infection Dynamics of ATG8 in *Leishmania*: Balancing Autophagy for Therapeutics

**DOI:** 10.3390/molecules27103142

**Published:** 2022-05-13

**Authors:** Vrushali Guhe, Farah Anjum, Alaa Shafie, Md Imtaiyaz Hassan, Visweswara Rao Pasupuleti, Shailza Singh

**Affiliations:** 1 National Centre for Cell Science, NCCS Complex, Ganeshkhind, SPPU Campus, Pune 411007, India; vrushali.guhe@nccs.res.in; 2 Department of Clinical Laboratory Sciences, College of Applied Medical Sciences, Taif University, P.O. Box 11099, Taif 21944, Saudi Arabia; f2016anjum@gmail.com (F.A.); dr.alaa.shafie.tu@gmail.com (A.S.); 3 Centre for Interdisciplinary Research in Basic Sciences, Jamia Millia Islamia, Jamia Nagar, New Delhi 110025, India; mihassan@jmi.ac.in; 4 Department of Biomedical Sciences and Therapeutics, Faculty of Medicine & Health Sciences, Universiti Malaysia Sabah, Kota Kinabalu 44800, Sabah, Malaysia; 5 Department of Biochemistry, Faculty of Medicine and Health Sciences, Abdurrab University, Pekanbaru 28291, Riau, Indonesia; 6 Centre for International Collaboration and Research, Reva University, Rukmini Knowledge Park, Katti-Genahalli, Yelahanka, Bangalore 560064, India

**Keywords:** Leishmaniasis, ATG proteins, mutational studies, conservation of ATG proteins, phylogeny, SCA, MD simulations, docking, thiabendazole

## Abstract

In many regions of the world, Leishmaniasis is a cause of substantial mortality and ailment. Due to impediment in available treatment, development of novel and effective treatments is indispensable. Significance of autophagy has been accentuated in infectious disease as well as in Leishmaniasis, and it is having capability to be manifested as a therapeutic target. By evincing autophagy as a novel therapeutic regime, this study emphasized on the critical role of ATG4.1-ATG8 and ATG5-ATG12 complexes in *Leishmania* species. The objective here was to identify ATG8 as a potential therapeutic target in *Leishmania*. R71T, P56E, R18P are the significant mutations which shows detrimental effect on ATG8 while Arg276, Arg73, Cys75 of ATG4.1 and Val88, Pro89, Glu116, Asn117, and Gly120 are interacting residues of ATG8. Along with this, we also bring into spotlight an enticing role of Thiabendazole derivatives that interferes with the survival mechanisms by targeting ATG8. Further, the study claims that thiabendazole can be a potential drug candidate to target autophagy process in the infectious disease Leishmaniasis.

## 1. Introduction

Leishmaniasis is an intricate disease proliferated by sand flies infected with *Leishmania* species. It is declared as the second most neglected disease, responsible for substantial morbidity and mortality [1]. Cutaneous Leishmaniasis (CL) is one of the medical manifestations of Leishmaniasis, which is characterized by its skin pathology such as crusted papules or ulcers on exposed skin [1,2]. Causative agents for CL are *Leishmania major*, *Leishmania tropica*, *Leishmania braziliensis*, *Leishmania mexicana*, and *Leishmania amazonensis* [2].

Leishmaniasis is a severe public health concern in many developing countries, but treatment options are limited. A cured original infection generally protects against secondary infections, meaning that a *Leishmania* vaccine should be simple to construct. Unfortunately, no effective vaccinations against this parasite are presently available in practice. As a result, chemotherapy is the most common method of sickness management. Pentavalent antimonials, amphotericin B, the oral medication miltefosine, and AmBiosome are among the few therapies available, all of which have substantial adverse effects [3]. Miltefosine and antimonials do not work for more than 60% of Leishmaniasis patients in Bihar (India), which is thought to be related to acquired resistance. The mechanisms of action and resistance linked to drug metabolism, thiol metabolism, and drug efflux have just been established in the last decade, although this class of medications has been used to treat Leishmaniasis for over 60 years. With the advent of new medicines such as miltefosine in 2002 and paromomycin in 2005–2006, it is necessary to design a plan to avoid the formation of drug resistance; combination therapy, therapy monitoring, and enhanced diagnostics might all play key roles in this approach [3,4]. Current medications for the treatment of Leishmaniasis have effectiveness and safety limits, necessitating the development of new therapeutic techniques. Evolution for drug discovery is a cardinal approach for the treatment of Leishmaniasis and these types of drugs can be proven as overriding candidates for medications for these neglected diseases.

Various studies highlighted on numerous techniques of *Leishmania* parasite by which it able to escape the immune system and resist host microbicidal actions and this can be a major reason behind developing resistance against available therapeutics. In host, parasite predominantly targets macrophages among diverse phagocytic cells [2,5]. The parasite stimulates the phagocytic route, which communicates with the autophagic system, and this contact improves the microbicidal processes involved in the innate host immune response, as it targets phagocytic cell macrophages. Autophagy is a cellular catabolic process that maintains cellular homeostasis and is conserved across the animal kingdom, from yeasts to mammals. Autophagy Related Proteins (ATGs) are involved in autophagic process. Even though new information is revealed from time to time, the mechanism of autophagy in humans and yeast is well understood [6]. So far, 41 different types of ATG proteins have been identified as playing a role in the autophagic process, which have been classified into different categories based on their function [7] and ATG1-kinase/ULK1 complex, phosphatidylinositol (PI) 3-kinase complex (PI3K), membrane protein ATG9, ATG2-ATG18 complex, ATG16L conjugation system, and ATG8 conjugation system are the six functional groups of ATG proteins [8,9,10,11]. Autophagy can be induced under a variety of circumstances, including starvation and stress conditions, such as infection. Autophagy has been found to encourage infection or facilitate parasite clearance by phagocytic process depending on parasite type and host history in recent decades; however, the evidence is conflicting [7].

In addition to humans, autophagic research is gaining fascination in protozoa, notably in the *Leishmania* parasite. Only few studies have attempted to investigate the functionality of autophagy in *Leishmania* infection [12,13]. *Leishmania* lifecycle progression is reliant on cellular remodeling during differentiation and the transition from promastigote to amastigote, with autophagy playing a vital role [13]. In comparison to humans, information on ATG proteins is limited, but studies on some ATG proteins in *Leishmania* have been highlighted. ATG5, ATG12, ATG8, ATG4, and ATG16 have been highlighted in the literature, with descriptions of their interactions and importance in parasite survival [14,15,16,17]. However, the exact mechanisms and phenomena involved in this process are unknown in the parasite. Here, we attempted to highlight the residual importance of amino acids for specific ATG proteins by considering its evolutionary conservedness with the other species especially with *Homo sapiens*. Even so, the central role of autophagy in Leishmaniasis attracts attention, and this phenomenon could be considered as a potential treatment option [12].

Thiabendazole is an antihelminthic benzimidazole derivative that inhibits the helminth-specific mitochondrial enzyme fumarate reductase, limiting the citric acid cycle, mitochondrial respiration, and consequent ATP synthesis, ultimately leading to helminth mortality [18,19]. Furthermore, thiabendazole has an overt ovicidal action on certain trichostrongylids and is thought to impede microtubule polymerization by binding to beta-tubulin. Thiabendazole has also been shown to be beneficial against parasitic infections such as strongyloidiasis and a rare cutaneous infection. Thiabendazole also has a function in cell division by binding to ß-tubulin and blocking polymerization, preventing the production of microtubules, and stopping cell division. In addition to this, it impairs glucose uptake leading to depletion of glycogen and stored ATPs, due to this energy-deprived conditions occur in the cell [20].

The limitations of currently available medications demand the development of new therapies, and the relevance of autophagy in Leishmaniasis positions it as a key target for therapeutic intervention [12]. Thiabendazole has antiparasitic properties and a function in glucose absorption and the ability to modify homeostasis via changing ATP storage in the cell [20]. Autophagy is a crucial subcellular process that aids in starvation and can also provide energy and nutrients to cells in a stressful situation. We hypothesized that as thiabendazole can target glucose absorption in cells, it may be shown as a superior way to battle Leishmaniasis by taking into account the importance of autophagy in parasite life [20]. Thus, in this study, we attempt to repurpose the available drug Thiabendazole for leishmaniasis and assess its efficacy in the parasite’s autophagic process.

Overall, we anticipate that our research will emphasize the important ATG proteins in parasites and how they might be used as a potential target in Leishmaniasis. In this study conservedness of autophagic process is taken into account as mentioned in literature [21]. We are trying to investigate if thiabendazole can target essential autophagic protein. Along with thiabendazole repurposing, we describe important ATG proteins, their interaction partners, and interacting residues. The current research also sheds light on the important amino acid from a specific protein, as well as any possible substitutions that may interfere with the protein’s normal functionality. Collectively, our study tries to emphasize that autophagy can be a novel target for disease treatment.

## 2. Methodology

### 2.1. Phylogenetic Tree Construction for Sequential Analysis

The elementary postulation for phylogenetic tree analysis is that all the sequences on a tree are descended from a common ancestor. This study pays attention to a phylogenetic tree constructed from molecular data of protein sequences, which indicated the conserved nature of ATG proteins in *Homo sapiens* and *Leishmania major*. Following are the distinct steps used to build a phylogenetic tree:

#### 2.1.1. Sequence Acquisition

The protein database of NCBI (National Center for Biotechnology Information, Bethesda, Maryland, United States) was employed to acquire sequences of ATG family proteins *Homo sapiens* and *Leishmania* major, further unique and non-iterative sequences were acquired in FASTA format [22].

#### 2.1.2. Multiple Sequence Alignment (MSA)

In phylogenetic analysis, MSA is a widely used bioinformatics approach in which the evolutionary tree is systematized in a hierarchical structure with closely related sequences kept together. Clustal Omega (EMBL’s European Bioinformatics Institute, EMBL-EBI, Cambridgeshire, UK) was used to perform MSA for all ATG proteins found in *Homo sapiens* and *Leishmania major* [22]. It uses a novel MSA algorithm that includes a guide tree and an HMM profile-profile technique to align three or more sequences. Default settings were used to set parameters and NEXUS was the output file format for aligned file. Acquired ATG proteins from NCBI were aligned using Clustal Omega [23].

#### 2.1.3. Phylogenetic Tree Construction

MrBayes v3.2.6 was availed to do Bayesian phylogenetic analysis; it read the aligned file in NEXUS format [24,25]. Programming sequences for certain generations were conducted using MCMC (Metropolis Coupled Markov Chain Monte Carlo), and the analysis was terminated when the average standard deviation of split frequency was within 0.01–0.05. After achieving split frequency, summarization of the tree was run with the diagnostics in the MCMC command [23,24]. The output of the program is in the form of a cladogram with the posterior probabilities for each split and a phylogram with mean branch lengths. The tree was visualized or read by Java-based application FigTree [25]. Phylogenetic tree reconstruction was used to check the conservedness among ATG proteins with respect to *Homo sapiens* and *Leishmania major* and also between the species.

### 2.2. Statistical Coupling Analysis (SCA) for Positional Conservedness Analysis

Statistical coupling analysis (SCA) is a technique for examining multiple sequence alignments that were used to categorize groups of co-evolving residues termed “sectors” namely blue, green, and red [26]. SCA essentially offer information regarding co-evolving positions, from which positional conservatism can be assessed. The aligned file feed is in the PEARSON format, and SCA was performed using the SCA toolbox in MATLAB R2020a (The MathWorks, Inc., Torrance, CA, USA) [26,27]. The location of amino acid residues at specific sectors was determined using Dij values, and the conservation of residues with regard to the position was estimated [26,28].

### 2.3. Structural and Functional Residue Validation

Structural and functional residues are essential for the functionality of protein, thus, identifying these residues which are conserved evolutionary is crucial. The ConSurf server [Available online: https://consurf.tau.ac.il/ (accessed on 19 May 2021)] [29] is a novel bioinformatics tool for estimating the evolutionary conservation of amino acids based on their structure and functionality. The conservation score of amino acids is based on the protein family’s evolutionary relationships with their homologue sequences. The server uses an empirical Bayesian algorithm or a maximum likelihood (ML) method to evaluate evolutionary rates [30]. Since a protein’s conservation score is heavily influenced by its structural and functional significance, it may reveal crucial amino acids for the protein’s structure and function.

### 2.4. Effect of Conserved Amino Acid Substitution on Protein

The protein functionality depends on conserved amino acid residues. Here we try to check the effect of substituting of essential amino acid residue on the functionality. Based on the findings, we hope to highlight residues of vital ATG proteins whose mutations will jeopardize Leishmania major survival. Furthermore, alterations of these critical residues may aid in the exploration of residual relevance in parasite survival and infectivity. To predict the effect of specific mutation we have used various web tools:

#### 2.4.1. SDM (Site Directed Mutator)

SDM is a web server [Available online: http://marid.bioc.cam.ac.uk/sdm2 (accessed on 12 June 2021)] [31] that is used to investigate the effects of mutations on protein stability and functionality. On the server, a 3D structure of the wild-type protein is required, and mutations are delivered in two formats: as a single mutation or as a mutation list. We used the mutation list option to assess the effect of each amino acid mutation on protein stability and predicted mutations that result in a decrease in protein stability after mutation [31].

#### 2.4.2. PROVEAN (Protein Variation Effect Analyzer)

PROVEAN is a web server [Available online: http://provean.jcvi.org/index.php (accessed on 15 June 2021)] [32] that demonstrates the effect of amino acid alterations on the functionality of the protein. Provean predicts the functioning of proteins from any species, using the protein sequence of interest as the input format and the required amino acid substitutions file as the output format, and it will produce a Provean score and apparently the prediction after each substitution on the protein [32].

#### 2.4.3. SODA

SODA is a web server [Available online: http://old.protein.bio.unipd.it/soda/help (accessed on 14 June 2021)] [33] that informs about the solubility of protein which is a crucial parameter that may alter after the mutations occurred. SODA is a computational method that predicts the changes of protein solubility based on the aggregation and disorder propensity by comparing the profiles of wild-type proteins. As insoluble regions in proteins aggregate, this may lead to cause various diseases; these mutations will give detrimental effect [33].

#### 2.4.4. Arpeggio

Arpeggio server [Available online: http://biosig.unimelb.edu.au/arpeggioweb/ (accessed on 15 June 2021)] [34] analyze the non-covalent interactions and calculates the number of interatomic interactions of a protein molecule. PDB structure of the protein is required by a server, it can calculate about 15 types of interactions of a protein. Through the server, comparison between wild type protein and mutated protein based on non-covalent interactions is possible [34].

### 2.5. Structure Prediction, Validation, and Interaction studies

3-Dimensional structures of ATG proteins from *Homo sapiens* and *Leishmania major* were modeled using Homology modeling by using Modeller9.23 [35] and Ab initio modeling by Robetta server [Available online: https://robetta.bakerlab.org/ (accessed on 2 March 2021)] [36]. The structures were visualized and evaluated using PyMOL v1.7.4.5 (originally developed by Delano Scientific LLC and now owned by Schrodinger, Inc) [37] and RAMPAGE. For the interactions’ studies, the HADDOCK web server [Available online: https://wenmr.science.uu.nl/haddock2.4 (accessed on 20 March 2021)] [38] is used, which highlights the protein-protein interacting residues. HADDOCK is a favored docking tool that allows creating data-driven docking, by utilization of known experimental data. Active residues from experimentally proven research were entered into HADDOCK for site specific docking [38]. It constructs the topology of the molecules to be docked automatically. ATG4-ATG8 complex and ATG12-ATG5 complex were docked by employing HADDOCK. The docking methodology is then subdivided into three steps: rigid-body energy minimization, semi-flexible refinement in torsion angle space, and explicit solvent refinement. The HADDOCK score will be assigned by a weighted combination of van der Waals, electrostatic, desolvation, and restraint violation energies plus buried surface area to each docking complex. ClusPro web server [Available online: https://cluspro.org (accessed on 1 April 2021)] [39] is also used for ATG16 complex interaction studies. Since interacting residues for ATG16 in relation to ATG5 and ATG12 are not reported in the literature, we have used the ClusPro online server for docking, which require protein in PDB format and active residues are not compulsory. It provides results in the form of clusters based on the low energy and RMSD score. PDBePisa is used to look for residues of amino acids on interfaces that are involved in interactions. The results were presented in the form of clusters, which were then shortlisted using PDB sum’s cluster analysis [40,41].

### 2.6. Virtual Screening

Virtual screening based on targets, combined with docking studies, enables for a larger data set of possible ligands to be searched at a lesser cost than traditional experimental screening. PubChem includes one of the largest corpuses of freely available chemical information; it has almost 157 million chemical substance descriptions contributed by depositors, 60 million unique chemical structures, and 1 million biological assay descriptions [42]. Based on literature findings, thiabendazole is thought to be a plausible medicinal molecule against parasite infection as well as a molecule that obstruct the cell’s glucose uptake metabolism [20].

Thiabendazole derivatives were explored and obtained for virtual screening from PubChem. Subsequently, significant cut-offs were applied to each of these structural databases, and all of the compounds were screened and filtered based on similarity, using Lipinski’s rule, a thumb rule for determining if a chemical compound has pharmacological or biological activity, it aids in discriminating between drug-like and non-drug-like compounds [43].

### 2.7. Molecular Docking

In structural molecular biology and computer-assisted drug design, molecular docking is a fundamental tool. The objective of ligand-protein docking is to predict a ligand’s most common binding modes with a three-dimensionally known protein. Methods for docking that work effectively towards search high-dimensional spaces and utilize a scoring formula that ranks candidate dockings accurately [44]. The AutoDock Vina tool 1.5.6 [45], molecular docking software with an easy-to-use interface for processing ligands and targets, as well as adding polar hydrogen atoms and Gasteiger charges, was utilized in this study. The predicted structure of ATG8 was utilized to dock against the screened thiabendazole derivatives; the positions with the lowest binding energy were analyzed and recorded in PDB format. The interaction of docked files was analyzed using LigPlot v.2.2 [46].

### 2.8. Molecular Dynamics Simulation

Stability and sustainability of proteins were performed using Molecular Dynamics Simulation by DESMOND 3.2 (D.E. Shaw Research, New York, NY, USA) [47]. All the proteins were simulated for 300 ns chemical time. Appropriate charges were added to neutralize ions in the system. For equilibration, TIP3P solvation system and NPT ensemble was used. The RMSD plot indicates the stability of proteins, quality and event analysis of protein was also performed [47].

## 3. Results

### 3.1. Conservedness Analysis of ATG Proteins

#### 3.1.1. Sequential Conservedness Analysis

**Figure 1:**
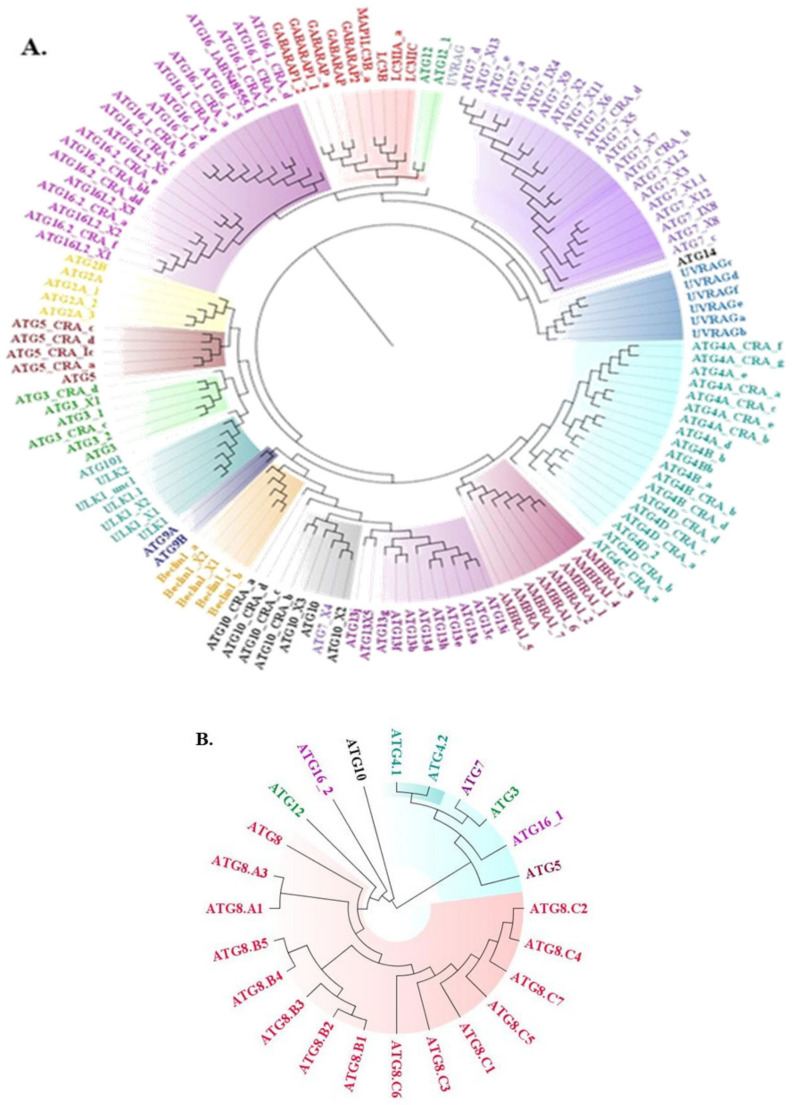
Phylogenetic tree illustrating evolutionary conservation across ATG proteins; different color keys indicate different types of ATG proteins. (**A**) Phylogenetic tree of *Homo sapiens* (**B**) Phylogenetic tree of *Leishmania major*.

**Figure 2:**
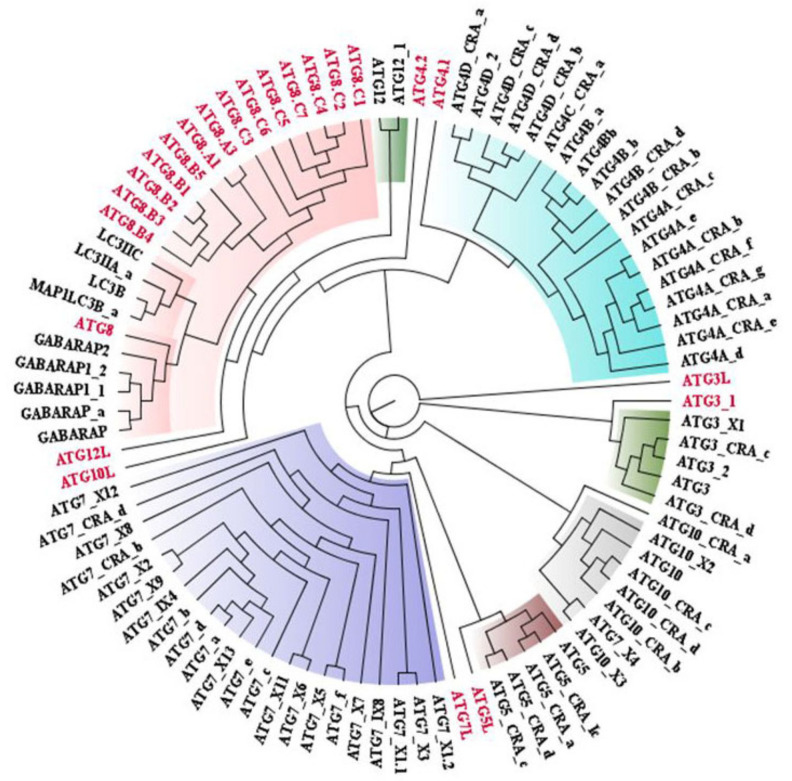
Phylogenetic tree showing similarity between ATG proteins among *Homo sapiens* and *Leishmania major* (Proteins showing black color annotation: *Homo sapiens* and Red color: *Leishmania major*).

ATG protein sequences which are directly playing role in the autophagic process were retrieved from the NCBI sequence database of *Homo sapiens* and *Leishmania major*. Collectively, 137 sequences were acquired from *Homo sapiens* precisely, ULK1, ATG3, ATG2, UVRAG, ATG7, ATG12, LC3, ATG16, ATG14, AMBRA, ATG4, ATG5, Beclin1, ATG13, ATG10, and ATG9. All accessible isoforms of these ATG proteins were retrieved (accession IDs of proteins mentioned in Supplementary Table S2). Phylogenetic tree analysis inferred that proteins are evolved from a common ancestor and annotated color for particular proteins denoted that the proteins evolved together (Figure 1). Similarly, 24 sequences were retrieved from *Leishmania major*, among which incidences of ATG3, ATG4, ATG5, ATG7, ATG8, ATG10, ATG12, and ATG16 occurred (accession IDs of proteins mentioned in Supplementary Table S1). Centered on the phylogenetic tree we infer that proteins are evolved together and closely related within the ATG family of *Homo sapiens* and *Leishmania major* (Figure 1). Since all proteins from *Homo sapiens* are not present in *Leishmania major*, we examined whether they are closely related or distinctly related and found that ATG proteins are evolutionarily related among *Homo sapiens* and *Leishmania major* (Figure 2). Therefore, from sequential conservedness analysis, we anticipated that while targeting autophagy in Leishmaniasis, conservedness among ATG proteins is to be considered. As sequential conservedness analysis shows similarity in *Homo sapiens* and *Leishmania major* ATG proteins, it is necessary to check for positional conservedness of ATG proteins in *Leishmania major* and whether those conserved residues show positional conservation in *Homo sapiens* ATG proteins or not. Since sequential similarities are there further positional conservation of ATG proteins were carried out.

#### 3.1.2. Positional Conservedness Analysis

**Figure 3:**
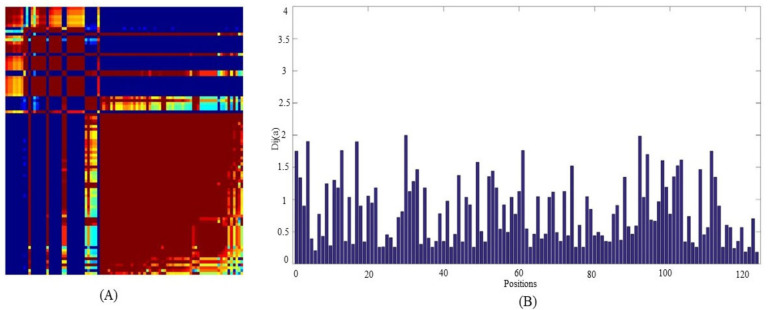
(**A**) Red, blue, and green sectors of a Statistical Coupling Analysis (SCA) matrix, with the red sector indicating highly conserved amino acid positions. (**B**) The conservedness score graph depicts the Dij value of a specific location.

As sequential conservedness is observed, for elucidating the positional conservedness among ATG proteins in *Leishmania major*, Statistical Coupling Analysis (SCA) was performed. It helps to quantitatively deduce the positional conservedness of protein sequences in the form of collectively evolving groups for conserved amino acid positions which are termed as “sectors”. For obtaining conserved positions among ATG proteins, sequences were trimmed with cutoff value 0.4 and sequences were trimmed accordingly to reduce redundant sequences before proceeding further for the analysis. Based on the Eigen value (Dij) score, residues were divided into three sectors namely Red, Blue, and Green which represents the conservation of position. Conservation order is increasing from Green, Blue, and Red. SCA depicts that most of the autophagic proteins are conserved according to positions in *Leishmania major* as majorly residues are lying in red sector (Figure 3). Sequential and positional conservedness analysis suggest that parasite and host are showing sequential conservedness and positional conservedness within species, depicting that ATG proteins are displaying conservedness among *Leishmania major* and *Homo sapiens* as well as in conservedness within *Leishmania major*.

### 3.2. Structural and Functional Residue Validation

**Figure 4:**
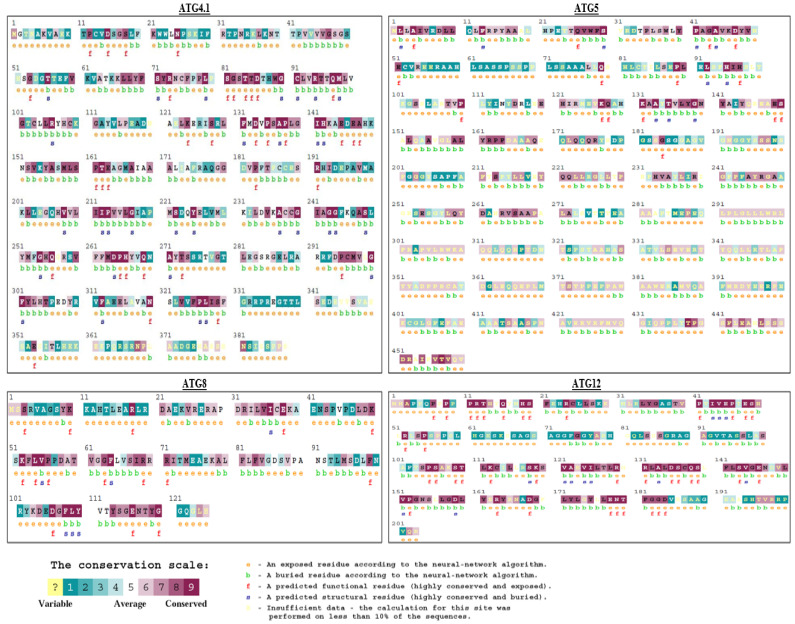
Sequential Conservation analysis of ATG proteins using ConSurf web server A. ATG12, B. ATG8, C. ATG4.1, D. ATG5.

As positional conservedness was observed within species, further, we have tried to examine amino acid residual conservation against the available proteins of all the species. Literature recommended that ATG8, ATG4.1, ATG5, and ATG12 are important proteins in *Leishmania major* in autophagic process [14,15,16,17]. Furthermore, we examined for residual importance of each protein concerning the evolutionary conservedness. ATG8 (XP_001682752.1), ATG4.1 (XP_001685715.1), ATG5 (CAJ06097.1), and ATG12 (XP_001683281.1) are acquired from NCBI and utilized for subsequent studies. ConSurf tool was used to study the conservation score of the specific position of amino acid using multiple sequence alignment. Based on the conservation score, amino acids’ structural and functional importance is speculated. The Bayesian method for Machine Learning is employed to calculate conservation score which ranges between 1 to 9. 1 denotes the least conserved position; while 5 is depicting the intermediate conservedness and ≥9 are showing high conservedness. Therefore, important amino acids from ATG proteins of *Leishmania major* were predicted for ATG8, ATG4.1, ATG5, and ATG12 (Figure 4). As conservation score implies, important functional and structural residues reflect the structure and function of the protein. Thus, the study here focusses on the possible mutations which can show a detrimental effect on the protein and ultimately affect the function and structure of the protein.

### 3.3. Effect of Conserved Amino Acid Substitution on Protein

Functional and structural residues are important for protein and its activity. In this study, we have tried to look into the effect of essential amino acid mutation on the functionality of the protein. Different online servers are available to illustrate the effect of point mutation on protein and we have tried to dissect the roles of the amino acids in ATG8, ATG4.1, ATG5, and ATG12 are the essential ones for the autophagic process. Accordingly, residues mutated with all the other amino acids and also on the basis of ΔΔG, mutations were highlighted with the help of the SDM server. ΔΔG is a unit to figure out how a single mutation can affect the stability of the protein. After mutation in the above four ATG proteins, specific amino acids were picked up for further validation. The main objective of this mutational study was to perceive amino acids which are showing deleterious effects; thus, those can be targeted for antileishmanial therapy by utilizing the autophagic machinery. Afterward, mutations which are providing stability to protein were sorted out. PROVEAN helps to understand its effect on the biological function of the protein. With the help of PROVEAN damaging mutations were shortlisted where scores less than −2.5 mutations were considered as deleterious, while a score greater than −2.5 were reviewed as neutral. Solubility of mutated protein is important for biological functioning, and here, the major goal is to manipulate parasitic protein in such a way that, its functionality hampers parasite survival. SODA calculates the solubility of protein variants based on aggregation propensity, disorder, helix, and strand propensity. Based on the above analysis, mutations were chosen (Table 1). In corroboration to this, the effect of mutation on its non-covalent interactions as compared to its wild-type structure is also calculated with the help of Arpeggio (Supplementary Table S3). Mutational studies have revealed that the residues listed in Table 1 can influence the protein’s specific functions and detrimental effect on proteins affecting the parasite’s survival.

### 3.4. Computational Structure Prediction, Validation and Molecular Dynamics Simulation

**Figure 5:**
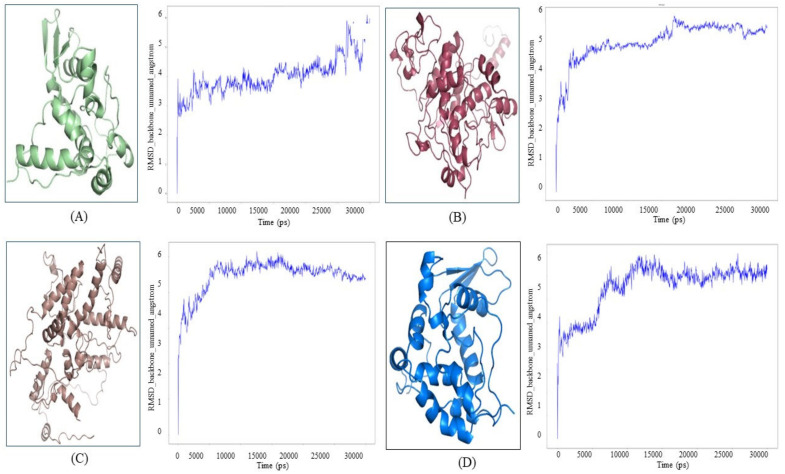
Structure Prediction of proteins and their respective Root mean square deviation (RMSD) plots for (**A**) ATG8 (**B**) ATG4.1 (**C**) ATG12 (**D**) ATG5 simulated for 300 ns.

ATG proteins of parasites are found to be conserved and co-evolved during the course of evolution; thereby we have checked for the structural availability. Analysis of evidence and known data confirmed that ATG proteins’ structures are not known in *Leishmania major*. Hence structure prediction for ATG proteins was performed employing available sequences. Homology modeling, a comparative technique was employed using Modeller. Based on the concept of two sequences having a high degree of similarity/identity, their structures are also similar. As a consequence, the sequence similarity of ATG8, ATG4.1, ATG5 and ATG12 were investigated. BLAST (blastp suite) was used to compare the sequence similarity and results obtained revealed ATG4.1, ATG5, and ATG12 not showing any sequential similarity. Thus, for structure prediction of these proteins ab initio modeling was carried out using the Robetta server. However, ATG8 had almost 50% sequences similarity when aligned through BLAST (blastp suite). Thereby, we examined two templates with X-ray structures for the query sequence, ATG8 (XP 001682752.1): 3H9D_A crystal structure of *Trypanosoma brucei* (sequence similarity 53.39%), as both *Leishmania major* and *Trypanosoma brucei* are trypanosomatids, and 1GNU_A (sequence similarity 48.21%) crystal structure of *Homo sapiens* GABARAP (GABA receptor-associated protein). Multi template modeling technique was employed. Afterwards, the structure prediction of all four ATG proteins validation was performed using PDBsum; which checks the stereochemical quality of a protein structure and thereby produces plots for analyzing its overall and residue-by-residue geometry along with the Ramachandran plot analysis. Resolution of protein structure with at least 2 angstroms and R factor not greater than 20 can be considered as good quality structure, for these measurements, it is expected to have more than 90% residues in the most favored region in the given structure. Predicted structures satisfying the above criteria were considered as good quality structures and scrutinized for further validation. Representative structures of autophagic proteins are depicted in Figure 5.

### 3.5. Molecular Dynamics Simulation of Proteins

Molecular Dynamics is a computationally exhaustive method for simulating the physiological conditions in which our protein exists in order to observe behavioral changes in them by mimicking the biological environment. For simulations NPT ensemble was used, while TIP3P solvent was utilized by maintaining pH as neutral, these conditions were opted to mimic the biological environment in order to examine the structural stability and sustainability of the particular protein. Root mean square deviation (RMSD) is used to measure the quantitative similarity of the atomic coordinates between the superimposed structures. It measures how much a protein conformation has changed throughout a complete production run. We have performed simulation for 300 ns in this study. RMSD of ATG4.1, ATG8, ATG12 and ATG5 are 12.186 Å, 5.965 Å, 7.228 Å, 8.78 Å, respectively. RMSD plots for respective structure are represented in Figure 5.

### 3.6. Protein-Protein interactions

**Figure 6:**
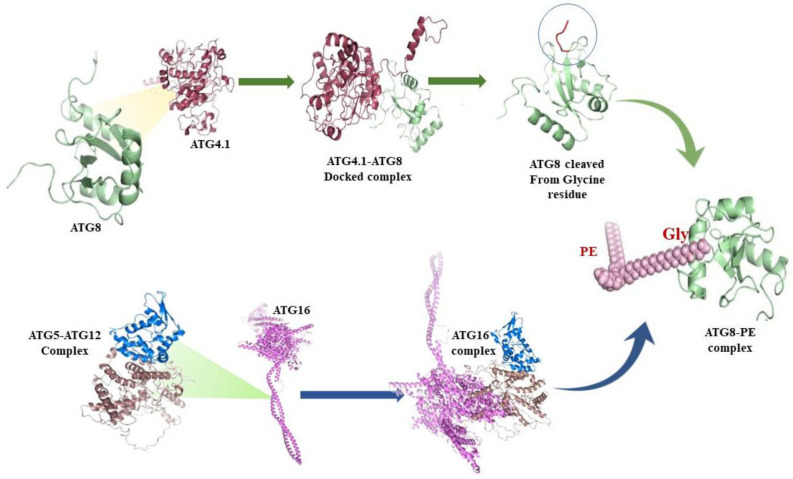
Autophagic proteins interacting with each other in different stages of autophagosome formation in *Leishmania major*.

Available literature suggests that ATG8 and ATG4.1 and ATG5 and ATG12 interact with each other and also the interacting residues essential for their particular interaction are depicted. Protein-protein complex interactions were studied utilizing available information to examine the other important residues for interactions, other than what was already reported in the literature HADDOCK server has used these interacting residues as active residues to interpret interfacial residues that are important in protein interaction. Further, it provided clusters for each interacting protein pair, for the ATG4.1-ATG8 complex there were 12 clusters and for the ATG5-ATG12 complex, there were 7 clusters provided. Clusters with zero residues in the disallowed region of the Ramachandran plot were confirmed for interfacial residual analysis through PDBePisa. The clusters with maximum interacting residues based on non-covalent analysis were chosen. PDBsum was used to analyze the interaction complex, which depicts essential amino acids for the specific interactions among ATG8 and ATG4.1 and ATG5 and ATG12 are illustrated in Table 2. Crucial residues accountable for the interaction of ATG proteins are Arg276, Arg73, Cys75 of ATG4.1 are interacting with Val88, Pro89, Glu116, Asn117, Gly120 of ATG8. Similarly, Glu125, Pro269, Lys131, Arg146, Gln128, Lys127, Tyr115, Gln175 of ATG5 interacts with Arg186, Lys29, Glu191, Glu45, Glu22, Gly182, His80, Arg79 of ATG12. ATG5-ATG12 complex further interacts with ATG16 protein to form the ATG16 complex (Figure 6), which is required for ATG8 stability on the autophagosome membrane. Interaction studies revealed that ATG12 interacts with ATG5, and ATG5 interacts with ATG16 protein (Table S4). ClusPro was used to dock ATG5, ATG12 and ATG16 complex; as a result, 100 clusters were obtained. Based on Ramachandran plot analysis and interfacial residual analysis ATG16 complex was finalized. As a result, we can conclude that ATG5 is an important protein for ATG8 stabilization.

### 3.7. ATG8 Can Be a Novel Target for Therapeutics against Leishmaniasis

**Figure 7:**
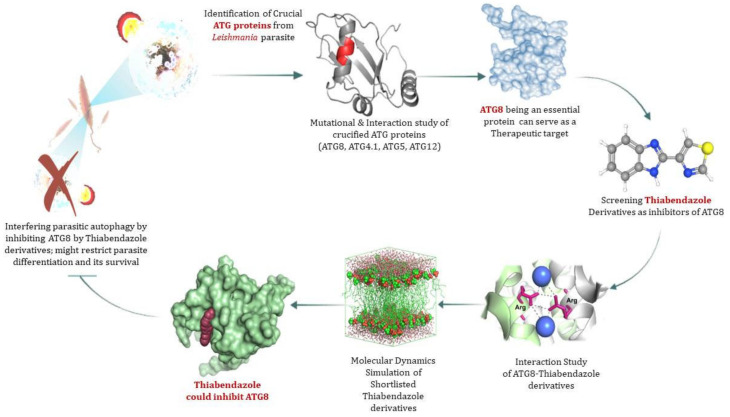
Identification of target protein ATG8 from *Leishmania major* and screening of Thiabendazole derivatives against ATG8.

ATG8 being a critical protein for parasite development and infectivity, is also regarded as an autophagic marker and as a result being investigated as a potential therapeutic target. Data suggests, it can be concluded that the interaction of the ATG16 complex is required for the activation of ATG8 and its proper recruitment to the autophagosome. Mutational studies have emphasized essential amino acid residues of ATG8 for its functionality. While, as previously stated, Thiabendazole has the potential to be a viable pharmacological target against Leishmaniasis [18,19]. Thus, this study hypothesized that Thiabendazole could act as a drug by targeting ATG8 as a novel therapeutic target for Leishmaniasis by targeting the parasite’s autophagy process. Graphical representation of targeting ATG8 against Leishmaniasis by using Thiabendazole lead as a drug molecule is depicted in Figure 7.

### 3.8. Virtual Screening of Thiabendazole Derivatives against ATG8

Thiabendazole was previously used against cutaneous and Leishmanial infection [18,19]. Derivatives of Thiabendazole were acquired from PubChem, total of 758 similar structures of Thiabendazole were downloaded from PubChem in 2D and 3D format. Physical screening of extensive libraries of chemicals against a biological target is the most common method for identifying novel lead compounds in drug discovery. Virtual screening is an alternate strategy that involves computationally screening vast libraries of chemicals for compounds that complement known structural targets and then testing those that are anticipated to bind well empirically. The Lipinski Rule of five was used to identify potential targets of Thiabendazole derivatives for drug-like property. Lipinski’s Rule of Five suggests drug molecules should have a molecular mass less than 500 Dalton, high lipophilicity (LogP) less than 5, hydrogen bond donors less than 5, hydrogen bond acceptors less than 10, molar refractivity should be between 40–130. Based on this rule of five, 758 similar structures of Thiabendazole were further scrutinized and final screening was carried out on the basis of molecular docking of derivatives against ATG8.

### 3.9. Molecular Docking and Molecular Dynamics Simulation (MDS)

**Figure 8:**
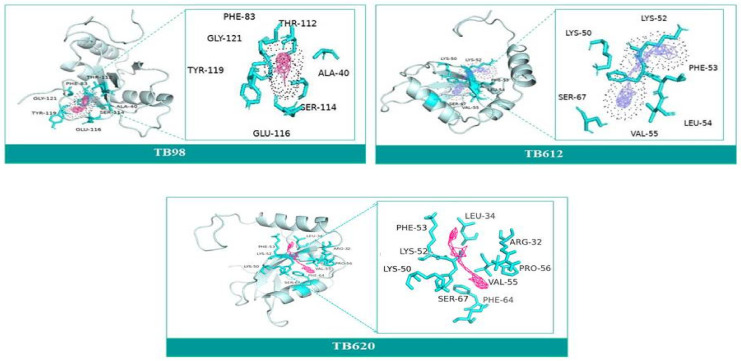
Top 3 Hits of Thiabendazole Derivatives with their interacting residues and Docked Conformations.

**Figure 9:**
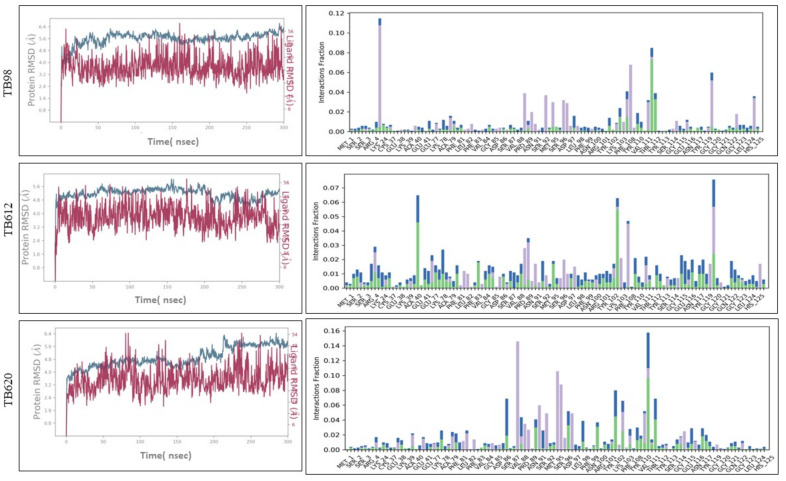
Root Mean Square Deviation (RMSD) Plots and Protein-Ligand Interaction Plots of ATG8 and top three shortlisted Thiabendazole derivatives.

The goal of molecular docking is to predict ligand-binding sites on receptor surfaces as well as the binding affinities associated with them. Shortlisted derivatives of Thiabendazole were docked blindly with the predicted structure of ATG8 and virtually screened on the basis of molecular docking. Each docking had nine docked conformations assigned to it and the conformation of derivatives with the lowest binding energy was chosen for molecular dynamics simulations. Molecular docking had screened library to top 10 hits and their respective binding energies, interacting residues are mentioned in Supplementary Table S5, and docked conformations are represented in Supplementary Figure S1. To further investigate the interplay of amino acids and derivatives, LigPlot v.2.2 was used. Dominant three derivatives among ten were concluded on the basis of molecular dynamics simulation studies and related details are enlisted in Table 3. Root Mean Square Deviation (RMSD) plot delineates stability of interacting Thiabendazole derivatives with ATG8. Docked conformations and RMSD plots and interacting residues graphs of the top three hits are represented in Figure 8 and Figure 9, respectively.

## 4. Discussion

Developed resistance and severe side effects to the available drugs, have suggested that a potential therapeutic regime against Leishmaniasis is urgently needed. In order to develop therapeutics, the discovery of novel targets is a preliminary goal [18]. Autophagy is a target for therapeutics in infectious diseases because it maintains cellular homeostasis and also plays an important role in cellular metabolism [48]. The *Leishmania* parasite can induce autophagy in host cells, as previously highlighted in literature shows mammalian autophagic proteins LC3 in murine macrophages after infection [12]. Studies also confirmed that parasites regulate autophagy in a time-dependent manner after infection [12]. This resulted in the conclusion that autophagy plays a role in the survival of *Leishmania* inside infected macrophages. Along with this, the importance of the parasite’s autophagic process is noteworthy. Autophagy is required for parasite differentiation and transformation from promastigotes to amastigotes form [16,49]. As the parasite’s survival inside the host is dependent on the evolutionary conserved autophagic process, this study focused on autophagy as a potential target for novel therapeutic interventions to combat the disease [48].

Since various ATG proteins have been reported in both the host and the parasite, we retrieved all available ATG protein sequences from NCBI for both *Homo sapiens* and *Leishmania major*. Considering evolutionary conservedness, our study primarily is focused on conservation among ATG protein of *Homo sapiens* and *Leishmania major* [21]. Phylogenetic analysis interpreted that ATG proteins evolved together within the species, and also showed similarity among ATG proteins between the species. As a result, we have examined the positional conservedness of amino acids in ATG proteins, and the results clearly indicate positional conservedness. Thus, based on positional conservation, we attempted to identify functional and structurally important residues in *Leishmania major* ATG proteins. Since functionally and structurally important residues are important for proteins, we tried to mutate particular residues computationally and predict their effect on protein functionality. This study emphasized the importance of pertinent residues and their possible replacement by the other amino acids, demonstrating the detrimental effect on the protein. Previously, studies also reported that ATG4.1 has conserved catalytic triad comprising of Cys73, His241, and Asp239 while C-terminal region of ATG8 incorporates Leu159, Val209, Cys296, and C-terminus of ATG8 is cleaved by ATG4.1, in which Leu54, Phe81, and Phe83 are found to be conserved in *Leishmania major* as compared to other species [14,16]. Our results explicated important residues of ATG4.1 (D113T, P213G, G244I, P265K), ATG8 (R71T, P56E, R18P), ATG5 (P110C, P89K, D47P), and ATG12 (P41K, P152N, E178T, N179R) which have detrimental effect after mutations. The results clearly show that we excluded the conserved residues among ATG proteins while using them in the mutational study and the results illustrated the essential amino acid residues whose mutation will have an impact on the protein’s existing functionality.

In addition to this, we attempted to shed light on the interaction of ATG proteins, as these interactions are critical for the activation of the autophagic process. ATG4.1 interacts with ATG8, Leu159, Val209, and Cys296 residues from the C-terminus of ATG8 are cleaved by Leu54, Phe81, and Phe83 of ATG4.1 [16]. Since there are few known residues that play role in interactions, we tried to focus on more residues that are important for these interactions. The availability of protein structure is required for protein-protein interactions, which leads us to predict the structure of important autophagic proteins that play an important role in complex formation, and the proteins involved are ATG4.1, ATG8, ATG5, and ATG12 [14,15,16]. The stability of predicted structures was further confirmed through Molecular Dynamics simulation, and further interaction studies were performed. The result of the interaction study shows that these interactions are important for the activation of ATG8, which is an autophagic marker for autophagy. As the results indicated that ATG8 is important for the autophagic process, we attempted to study ATG8 at the molecular level in order to predict pivotal amino acid residues of ATG8. It is also reported that in *Leishmania donovani* parasite requires Atg8 protein for infectivity and survival under stress [15]. Since literature evidence and results indicated that ATG8 is playing a pivotal role, so for a detailed study ATG8 was considered [15]. The interaction of ATG8 with its interacting partner was undertaken in order to identify all the important residues of ATG8. In addition to ATG8, we have mentioned important residues for ATG4.1, ATG5, and ATG12. Arg276, Arg73, Cys75 of ATG4.1 and Val88, Pro89, Glu116, Asn117, and Gly120 of ATG8 are crucial interacting residues for ATG4.1-ATG8 interaction. Similarly, Glu125, Pro269, Lys131, Arg146, Gln128, Lys127, Tyr115, Gln175 of ATG5, and Arg186, Lys29, Glu191, Glu45, Glu22, Gly182, His80, Arg79 of ATG12 are playing important role in ATG5-ATG12 interaction. Overall, conservation study suggests that ATG proteins are similar in host and parasite, and positional conservation was also observed. Thus, if we want to target specific autophagy proteins, it is essential to understand which amino acid residues are functionally active, as specifically targeting the ATG proteins of *Leishmania major* is providing utmost importance to therapeutic intervention studies. As a result, the study is focused on all important residues that are important for protein by taking into account protein structure and its interacting tendency. Summarizing this, study is helpful to understand important ATG proteins and also emphasize on essential residues for its structure, function, interaction properties.

Furthermore, we tried to find a better drug option by examining the availability of currently available drugs. Thiabendazole is successfully administered antiparasitic drug known for showing effect on glucose uptake metabolism, and cell cycle [20]. Thus, taking it as a better option for combating Leishmaniasis by targeting the autophagic process, we have tried to test its effect on the important autophagic protein ATG8. We retrieved Thiabendazole derivatives and docked those derivatives with ATG8. Thiabendazole, based on docking analysis, has the potential to be a novel drug that targets autophagy. It can be said that in addition to the available drug options, we can test the effect of Thiabendazole derivatives on Leishmaniasis individually and possibly in combination.

Cumulatively, we are attempting to focus on persuading targets for combating Leishmaniasis. Despite the fact that we have used available experimental evidence to obtain the results, our entire study is based on computational approaches and experimental validation of this hypothesis is critical. In addition to this, the fact that the study does not focus on experimental data, it is able to highlight various unknown facts, such as the interactions of ATG8, ATG4.1, ATG5, ATG12, and ATG16 with their respective partners. In addition, investigate the ability to cornerstone the essential amino acid of ATG proteins, as well as their possible substitution, which may have a negative effect on protein activity. In addition, it is laying an emphasis on Thiabendazole as a potential target for Leishmaniasis and virtual screening pinnacled top three hits of Thiabendazole derivatives that may further be used. Again, in order to prove Thiabendazole as a drug, it is imperative to examine its cytotoxicity and physical effect on parasites, but this may serve as a concrete hypothesis for therapeutic interventions.

**Figure 10:**
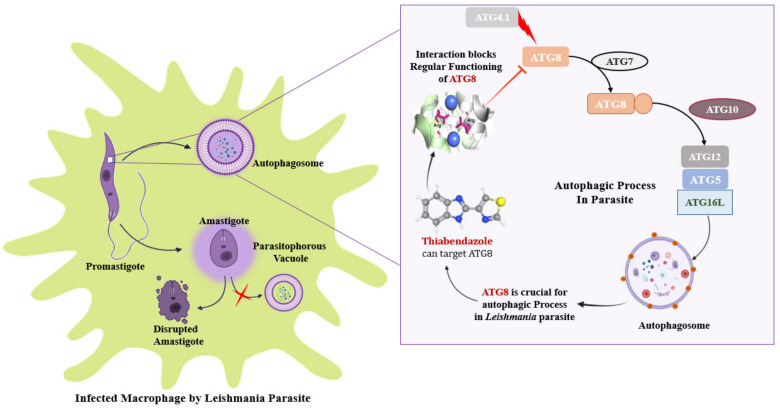
Illustrating the importance of ATG8 in *Leishmania* parasite, which is crucial for autophagic process. After binding with the Thiabendazole molecule, regular functioning of ATG8 can get hampered.

In nutshell, the research highlights that autophagy is crucial for the *Leishmania* parasite’s survival as well as cellular remodeling and differentiation. ATG8, ATG4.1, ATG5, and ATG12 are essential proteins for establishing a successful autophagic process in parasites, whereas, ATG8 plays an important function in diversified ways, including assistance in the formation of autophagosomes and also serving as an autophagic marker [49]. As a result, targeting ATG8 for a novel treatment approach can help to resolve disease wherein thiabendazole derivatives can be a potent drug molecule that can disrupt ATG8 functionality. Along with this, there are significant amino acid residues of ATG8 viz., Val88, Pro89, Glu116, Asn117, and Gly120 playing a key role in its structural stability while Leu159, Val209, Cys296 are responsible for its interacting activities. Along with ATG8, in this paper essential amino acid residues of ATG4.1, ATG5, and ATG12 have been highlighted. Hence it can be postulated that focusing on particular residues could lead to tailored therapy by inhibiting autophagy in the *Leishmania* parasite and ATG8 can be a novel target for therapeutic intervention by considering its importance in the autophagic process (Figure 10).

## 5. Conclusions

This study focused on repurposing the existing drug thiabendazole against Leishmaniasis by targeting the essential metabolic process autophagy. ATG8 is an important autophagy protein and targeting ATG8 could serve as an effective anti-Leishmaniasis treatment regimen. Thiabendazole derivatives namely N-(1H-Benzimidazol-2-yl)-4,5,6,7-tetrahydro-[1,3]thiazolo[5,4-c]pyridin-2-amine,1-[3-(1H-Benzimidazol-2-yl)propyl]-2-methyl-3-[[4-(trifluoromethyl)-1,3-thiazol-2-yl]methyl]guanidine, 2-[3-(1H-Benzimidazol-2-yl)propyl]-1-ethyl-3-[2-[4-(trifluoromethyl)-1,3-thiazol-2-yl]ethyl]guanidine could serve as novel drug candidates. Since ATG8 is a critical ATG protein found in the parasites, this study sheds light on its residual importance, functional and structurally important residues, and possible replacements of ATG8 are R71T, P56E, and R18P, all of which have an adverse influence on the protein. Along with this, interacting residues are also highlighted viz., Val88, Pro89, Glu116, Asn117, and Gly120. Overall, this study focuses on identifying ATG8 as a potential target in *Leishmania* that could be used as a point of intervention to address parasite survival facilitated by autophagy; highlighting an appealing role of thiabendazole derivatives that may interrupt with the survival machinery by targeting ATG8.

## Figures and Tables

**Table 1:** Amino acid substitution due to a specific mutation and its impact on Gibbs free energy (ΔΔG) and ultimately on protein stability, Protein Variation Effect Analyzer (PROVEAN) score reflects the functional effect of amino acid changes and their impact on protein functionality, SODA predicts the impacts of sequence variations on protein solubility.

	Amino Acid Substitution	ΔΔG	Stability	PROVEAN Score	Impact on Protein	SODA Score	Solubility
ATG8	R71T	−1.94	Decrease	−5.825	Deleterious	−14.878	Less Soluble
	P56E	−1.07	Decrease	−7.694	Deleterious	−2.13	Less Soluble
	R18P	−3.81	Decrease	−6.641	Deleterious	−1.685	Less Soluble
ATG5	P110C	0.29	Increase	−6.250	Deleterious	−50.412	Less Soluble
	P89K	−1.04	Decrease	−7.000	Deleterious	−2.053	Less Soluble
	D47P	−0.74	Decrease	−7.000	Deleterious	−2.279	Less Soluble
ATG12	P41K	−1.04	Decrease	−3.098	Deleterious	−60.883	Less Soluble
	P152N	0.91	Increase	−6.968	Deleterious	−13.018	Less Soluble
	E178T	−2.00	Decrease	−5.600	Deleterious	−15.209	Less Soluble
	N179R	−1.99	Decrease	−5.200	Deleterious	−1.375	Less Soluble
ATG4.1	D133T	0.03	Increase	−6.980	Deleterious	−12.363	Less Soluble
	P213G	−1.13	Decrease	−4.467	Deleterious	−213.32	Less Soluble
	G244I	−2.29	Decrease	−10.00	Deleterious	−17.447	Less Soluble
	P265K	−0.88	Decrease	−7.987	Deleterious	−11.794	Less Soluble

**Table 2:** ATG4.1-ATG8 and ATG5-ATG12 complexes interacting residues, as well as the type of interaction between interacting amino acids.

	ATG4.1	ATG8	Interactions
Interacting Residues	Arg276	Val88	Hydrogen Bond
	Arg276	Pro89	Hydrogen Bond
	Arg73	Glu116	Hydrogen Bond and Salt Bridge
	Arg73	Asn117	Hydrogen Bond
	Cys75	Gly120	Hydrogen Bond
	ATG5	ATG12	Interactions
Interacting Residues	Glu125	Arg186	Hydrogen Bond and Salt Bridge
	Pro269	Lys29	Hydrogen Bond
	Lys131	Glu191	Hydrogen Bond and Salt Bridge
	Arg146	Glu191	Hydrogen Bond and Salt Bridge
	Arg146	Glu45	Hydrogen Bond and Salt Bridge
	Glu128	Asp184	Hydrogen Bond
	Lys127	Glu22	Hydrogen Bond and Salt Bridge
	Tyr115	Gly182	Hydrogen Bond
	Gln175	His80	Hydrogen Bond
	Gln175	Arg79	Hydrogen Bond

**Table 3:** Screened Thiabendazole derivatives against ATG8 representing binding energies of ATG8-thiabendazole derivatives, structure and IUPAC names, molecular weight, LogP values, number of Hydrogen donor and acceptors of derivatives.

Derivatives	IUPAC Name	Structure of Derivative	Binding Energy	Molecular Weight	LogP	H-Donor	H-Acceptor
TB98	N-(1H-Benzimidazol-2-yl)-4,5,6,7-tetrahydro-[1,3]thiazolo[5,4-c]pyridin-2-amine	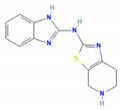	−8.1	271.34	2.2	3	5
TB612	1-[3-(1H-Benzimidazol-2-yl)propyl]-2-methyl-3-[[4-(trifluoromethyl)-1,3-thiazol-2-yl]methyl]guanidine	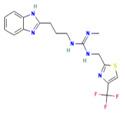	−8.4	396.4	2.9	3	7
TB620	2-[3-(1H-Benzimidazol-2-yl)propyl]-1-ethyl-3-[2-[4-(trifluoromethyl)-1,3-thiazol-2-yl]ethyl]guanidine	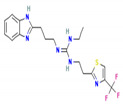	−8.6	424.5	3.7	3	7

## Data Availability

The data that support the findings of this study are available in the Supplementary Materials.

## Supplementary Materials

The following supporting information can be downloaded at: https://www.mdpi.com/article/10.3390/molecules27103142/s1. Table S1: ATG Proteins of *Leishmania major*; Table S2: ATG Proteins of *Homo sapiens*; Table S3: Binding Energies and Interacting Residues of top 10 hits; Table S3: Non-covalent Interactions of Protein Molecule; Table S4: Interacting Residues among ATG5 and ATG16.

## Sample Availability

Samples of the compounds are not available from the authors.

## Abbreviations

ATG	Autophagy Related Proteins
ULK1	unc-51-like kinase 1
PI3K	phosphatidylinositol (PI) 3-kinase complex
VPS	Vacuolar Protein Sorting
WIPI	WD repeat domain phosphoinositide interacting protein 2
DFCP1	Double FYVE containing protein 1
LC3	Microtubule-associated proteins 1A/1B light chain 3
SCA	Statistical Coupling Analysis
SDM	Site Directed Mutator
PROVEAN	Protein Variation Effect Analyzer
SCA	Statistical Coupling Analysis
PE	Phosphatidylethanolamine
