# Supplementary material for: Infection Dynamics of ATG8 in Leishmania: Balancing Autophagy for Therapeutics
Source: Molecules. 2022 May 13;27(10):3142. doi: 10.3390/molecules27103142 (PMC9147918; doi:10.3390/molecules27103142)
Supplement: Supplementary file 1 [file molecules-27-03142-s001.zip › molecules-1633645-supplementary.pdf]

Supplementary Material:

**Supplementary Table S1: ATG Proteins of *Leishmania major***	
**ATG protein**	**Accession ID**
ATG3	XP_001685756.1
ATG4.1	XP_001685715.1
ATG4.2	XP_001684616.1
ATG5	CAJ06097.1
ATG7	XP_001680904.1
ATG8.A1	XP_001682661.1
ATG8.A3	XP_001682667.1
ATG8.B1	XP_001682659.1
ATG8.B2	XP_001682662.1
ATG8.B3	XP_001682668.1
ATG8.B4	XP_001682669.1
ATG8.B5	XP_001682671.1
ATG8.C1	XP_001681178.1
ATG8.C2	XP_001681179.1
ATG8.C3	XP_001681181.1
ATG8.C4	XP_001681185.1
ATG8.C5	XP_001681187.1
ATG8.C6	XP_001681189.1
ATG8.C7	XP_001681190.1
ATG8	XP_001682752.1
ATG10	XP_001685302.1
ATG12	XP_001683281.1
ATG16_1	XP_001681499.1
ATG16_2	XP_001685545.1

Supplementary Table S2: ATG Proteins of *Homo sapiens*					
**ATG Proteins**	**Accession ID**	**ATG Proteins**	**Accession ID**	**ATG Proteins**	**Accession ID**
ATG13a	NP_001192048.1	ATG7_a	NP_001336161.1	GABARAP_a	AEZ06293.1
ATG13f	NP_001192050.1	ATG7_e	NP_001336166.1	LC3IIA_a	NP_115903.1
ATG13c	NP_001192049.1	ATG7_c	NP_001138384.1	GABARAP	NP_009209.1
ATG13d	NP_001333266.1	ATG7_b	NP_001129503.2	ATG16.2_CRA_f	EAW74879.1
ATG13i	NP_001333286.1	ATG7_f	NP_001336167.1	ATG16.2_CRA_e	EAW74878.1
ATG13j	NP_001333289.1	ATG7_X2	XP_024309080.1	ATG16.2_CRA_c	EAW74876.1
ATG13g	NP_001333284.1	ATG7_IX8	XP_016861038.1	ATG16.2_CRA_a	EAW74874.1
ATG13b	NP_001333247.1	ATG7_X6	XP_016861037.1	ATG16.1_CRA_b	EAW71040.1
ATG13e	NP_001333269.1	ATG7_X3	XP_016861032.1	ATG16.1_CRA_e	EAW71037.1
ATG13h	NP_001192051.1	ATG7_X1.1	XP_016861031.1	ATG16.1_CRA_d	EAW71036.1
ATG13X5	XP_016874107.1	ATG7_X8	XP_011531587.1	ATG16.1_CRA_c	EAW71035.1
ATG101	NP_068753.2	ATG7_X7	XP_011531586.1	ATG16.1_CRA_a	EAW71033.1
ATG14	FAA00433.1	ATG7_IX4	XP_011531583.1	ATG16.2_CRA_dd	EAW74877.1
UVRAGf	NP_001273695.1	ATG7_X1.2	XP_011531580.1	ATG16.2_CRA_bb	EAW74875.1
UVRAGd	NP_001273693.1	ATG7_X13	XP_024309083.1	ATG16.1_CRA_f	EAW71039.1
UVRAGe	NP_001273694.1	ATG7_X12	XP_024309082.1	ATG16_1	ABN48555.1
UVRAGa	NP_079389.2	ATG7_X11	XP_016861040.1	ATG16_1_6	NP_001350671.1
UVRAGc	NP_001273692.1	ATG7_X9	XP_016861039.1	ATG16_1_4	NP_001177195.1
UVRAGb	NP_001273691.1	ATG7_X5	XP_016861036.1	ATG16L2_X3	XP_011543634.1
UVRAG	Q9P2Y5.1	ATG7_CRA_b	EAW64097.1	ATG16L2_X5	XP_006718796.1
Beclin1_c	NP_001300929.1	ATG7_CRA_d	EAW64096.1	ATG16L2_X2	XP_006718795.1
Beclin1_b	NP_001300928.1	ATG10	NP_001124500.1	ATG16L2_X1	XP_005274433.1
Beclin1_a	NP_001300927.1	ATG10_X2	XP_016865433.1	ATG16_1_5	NP_001177196.1
Beclin1_X2	XP_016880752.1	ATG7_X4	XP_011541963.1	ATG3_2	NP_001265641.1
Beclin1_X1	XP_016880751.1	ATG10_X3	XP_011541962.1	ATG3_1	NP_071933.2
ULK1	NP_003556.2	ATG10_CRA_d	EAW95886.1	ATG3_X1	XP_011511376.1
ULK1_X2	XP_011537101.1	ATG10_CRA_c	EAW95885.1	ATG3_CRA_d	EAW79671.1
ULK1_X1	XP_011537100.1	ATG10_CRA_b	EAW95884.1	ATG3_CRA_c	EAW79670.1
ULK1.1	AAC32326.1	ATG10_CRA_a	EAW95882.1	ATG3	AAH02830.1
ULK1_unc1	EAW98529.1	ATG5	AGC52703.1	ATG12	ACD74941.1
ULK2	NP_001136082.1	ATG5_CRA_Ic	EAW48417.1	ATG12_1	NP_004698.3
AMBRA1_5	NP_001373940.1	ATG5_CRA_a	EAW48414.1	ATG2A	AAI10651.1
AMBRA1_1	NP_001254711.1	ATG5_CRA_c	NP_001273037.1	ATG2B	NP_060506.6
AMBRA1_4	NP_001287660.1	ATG5_CRA_d	NP_001273040.1	ATG2A_2	NP_001354900.1
AMBRA1_3	NP_001254712.1	GABARAP1_2	NP_113600.1	ATG2A_3	NP_001354901.1
AMBRA1_2	NP_001354400.1	GABARAP2	NP_009216.1	ATG2A_1	NP_055919.2
AMBRA1_6	NP_001354398.1	GABARAP1_1	NP_001350527.1	ATG9A	EAW70707.1
AMBRA1_7	NP_001354399.1	LC3B	NP_073729.1	ATG9B	NP_001303985.1
AMBRA	ABI74670.1	MAP1LC3B_a	AEZ06292.1	ATG4C_CRA_a	EAX06580.1
ATG7_d	NP_001336165.1	LC3IIC	NP_001004343.1	ATG4A_CRA_g	EAX02696.1

Supplementary Table S2: ATG Proteins of *Homo sapiens*	
**ATG Protein**	**Accession ID**
ATG4A_CRA_f	EAX02695.1
ATG4A_CRA_e	EAX02694.1
ATG4A_CRA_c	EAX02693.1
ATG4A_CRA_b	EAX02690.1
ATG4A_CRA_a	EAX02689.1
ATG4Bb	NP_847896.1
ATG4D_CRA_d	EAW84119.1
ATG4D_CRA_c	EAW84118.1
ATG4D_CRA_b	EAW84117.1
ATG4D_CRA_a	EAW84116.1
ATG4B_CRA_d	EAW71281.1
ATG4B_CRA_b	EAW71279.1
ATG4D_2	NP_001268433.1
ATG4A_e	NP_001308219.1
ATG4A_d	NP_001308218.1
ATG4B_b	NP_847896.1
ATG4B_a	NP_037457.3

Supplementary Table S3: Non-covalent Interactions of Protein Molecule					
	**ATG4.1**	**D133T**	**G244I**	**P213G**	**P265K**
Total number of contacts	12610	12602	12614	12612	12662
VdW interactions	234	234	235	235	236
VdW clash interactions	434	433	438	438	445
Covalent interactions	0	0	0	0	0
Covalent clash interactions	0	0	0	0	0
Proximal interactions	11942	11935	11941	11939	11981
	**ATG5**	**P110C**	**P89K**	**D47P**	
Total number of contacts	12428	12432	12440	12426	
VdW interactions	247	247	247	247	
VdW clash interactions	457	457	457	458	
Covalent interactions	0	0	0	0	
Covalent clash interactions	0	0	0	0	
Proximal interactions	11724	11728	11736	11721	
	**ATG8**	**P56E**	**R18P**	**R71T**	
Total number of contacts	4163	4532	4467	4499	
VdW interactions	64	78	75	77	
VdW clash interactions	209	164	161	164	
Covalent interactions	0	0	0	0	
Covalent clash interactions	0	0	0	0	
Proximal interactions	3890	4290	4231	4258	
	**ATG12**	**P41K**	**P152N**	**E178T**	**N179R**
Total number of contacts	6827	6852	6836	6804	6832
VdW interactions	120	123	119	120	121
VdW clash interactions	265	271	265	267	266
Covalent interactions	0	0	0	0	0
Covalent clash interactions	0	0	0	0	0
Proximal interactions	6442	6458	6452	6417	6445

Supplementary Table S4: Interacting Residues among ATG5 and ATG16			
	**ATG5**	**ATG16**	**Interactions**
Interacting Residues	Glu376	His1237	Hydrogen Bond and Salt Bridge
	Arg239	His1237	Hydrogen Bond
	Arg239	Arg1156	Hydrogen Bond
	Arg239	Arg1155	Hydrogen Bond
	Tyr246	Glu1430	Hydrogen Bond
	Phe427	Ser1399	Hydrogen Bond
	Gly431	Ser1420	Hydrogen Bond
	Glu376	Arg1156	Salt Bridge
	Glu279	His1151	Salt Bridge
	Ile432	Asp1323	Hydrogen Bond

Supplementary Table S5: Binding Energies and Interacting Residues of top 10		
**Thiabendazole Derivatives**	**Binding Energy**	**Interacting Residues**
TB103	-9.1	Ala59, Ser67, Phe64, Leu54, Phe53, Lys50, Lys52, Arg32, Pro56, Val55
TB620	-8.6	Lys52, Leu34, Phe53, Arg32, Lys50, Pro56, Leu54, Val55, Ser67,Phe64
TB619	-8.4	Val55, Arg32, Lys50, Phe53, Lys52, Val128, Leu54
TB612	-8.4	Lys52, Leu54, Lys50, Val55,Ser67, Phe53
TB46	-8.3	Phe64, Gly63, Ser67, Arg32, Val55,Pro56, Lys50, Leu54, Phe53, Ala59
TB78	-8.2	Gly63,Ala59, Asp58, Pro56, Val55, Leu54, Phe53, Phe64, Ser67
TB618	-8.2	Thr112, Ser114, Glu116, Gly121,Asn42, Tyr119, Phe83, Ala40
TB104	-8.0	Ala59, Ser67, Phe64, Phe53, Leu54,Val55, Gly63, Pro56
TB98	-8.1	Glu116, Tyr119, Ala40, Phe83, Gly121, Thr112, Ser114
TB644	-8.1	Gly121, Glu41, Phe83, Tyr119,Ala40, Thr118, Glu116, Ser114, Gly120, Thr112

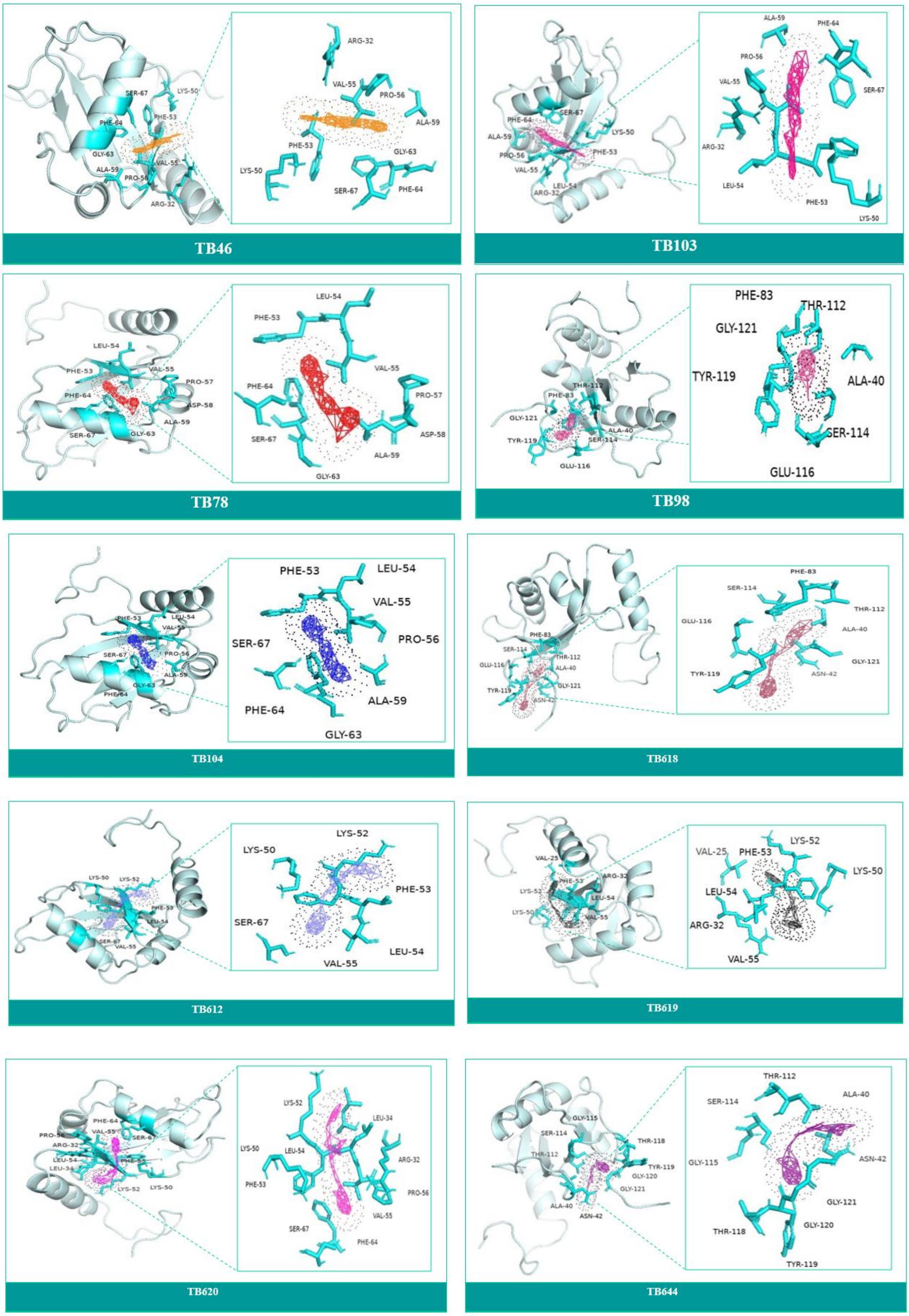

Supplementary Figure S1: Thiabendazole Derivatives with their interacting residues and Docked Conformations
